# Phospho-activated non-muscle myosin IIA heavy chain supports different mechanisms of virus uptake

**DOI:** 10.1128/jvi.00241-25

**Published:** 2025-11-11

**Authors:** Natalia Ansin, Kathia Guardado, Matías Ponce, María Paz García, Camila Ladra, Irene Ferreiro, Gonzalo Moratorio, Nicolás Sarute

**Affiliations:** 1 Laboratorio de Interacciones Virus-Célula, Institut Pasteur de Montevideo 123939, Montevideo, Uruguay; 2 Departamento de Inmunobiología, Facultad de Medicina, Universidad de la Republica 56724, Montevideo, Uruguay; 3 Burnett School of Biomedical Sciences, College of Medicine, University of Central Florida 6243, Orlando, Florida, USA; 4 Laboratorio de Evolución Experimental de Virus, Institut Pasteur de Montevideo 123939, Montevideo, Uruguay; 5 Laboratorio de Virología Molecular, Facultad de Ciencias, Universidad de la Republica Uruguay 56724, Montevideo, Uruguay; 6 Sección Genética Evolutiva, Facultad de Ciencias, Universidad de la Republica Uruguay 56724, Montevideo, Uruguay; Loyola University Chicago - Health Sciences Campus, Maywood, Illinois, USA

**Keywords:** virus entry, MYH9, proviral factor, tyrosine phosphorylation

## Abstract

Limiting virus entry is crucial to prevent infection, since this step of the virus life cycle is a major determinant of cellular tropism, host range, and pathogenesis. We recently showed that the transmembrane protein signal regulatory protein alpha (SIRPA), a negative regulator of F-actin-dependent phagocytosis, limits endocytosis of enveloped RNA viruses from unrelated families. Mechanistically, surface engagement of SIRPA recruits the SH2 domain-containing protein tyrosine phosphatases (SHP)-1 and -2, initiating a signaling cascade that dephosphorylates different pro-phagocytic proteins, including the heavy chain (MYH9) of the molecular motor non-muscle myosin IIA (NM-IIA), to ultimately suppress phagocytosis. Here, we analyzed whether MYH9 would enhance viral infection, as it does phagocytic engulfment, and how its activity in infection is regulated. We found that MYH9 expression increases the infection of viruses exploiting different endocytic pathways/mechanisms to enter cells, including flavivirus, arenavirus, rhabdovirus, and togavirus, in human and mouse cells. Furthermore, we demonstrated that MYH9 is actively translocated to the plasma membrane upon viral infection to support post-binding steps of entry, and that phosphorylation of two key tyrosine residues in its head and tail domains is essential for its function in viral infection. By using small molecule inhibitors and *MYH9* knockdown cells, we suggest that members of the Src family kinases may phosphorylate/activate MYH9 to support virus entry.

**IMPORTANCE:**

Viral infections represent a major threat to global public health and pose a huge social and economic cost; thus, a better understanding of how cell-intrinsic factors modulate the outcome of infection is of great importance to better understand virus–host interactions and to our ability to develop prophylactic measures and therapeutics. In this work, we show that MYH9 is a broad proviral host factor that enhances entry of divergent families of human pathogenic RNA viruses, which exploit different endocytic pathways to infect cells. By determining that virus infection triggers the phosphorylation of MYH9 in two tyrosine residues essential for its proviral activity, and the family of non-receptor tyrosine kinases involved in this process, we may also provide new cellular targets to develop antiviral therapies.

## INTRODUCTION

Viruses can hijack a variety of cellular processes and molecules to promote entry and replication in host cells (1). Although virus entry is a major determinant of cellular tropism, host range, and pathogenesis, there are relatively few host factors acting at this step whose activity has been characterized (2). We previously uncovered that signal regulatory protein alpha (SIRPA) is an intrinsic antiviral factor that limits entry of pathogenic enveloped RNA viruses from divergent families (3). SIRPA is a receptor-type transmembrane glycoprotein highly expressed on myeloid cells, harboring two immunoreceptor tyrosine-based inhibition motifs (ITIM) in its cytoplasmic domain (4). Phosphorylation of the SIRPA ITIMs provides docking sites for the recruitment and activation of the cytosolic SH2 domain-containing protein tyrosine phosphatases (SHP)-1 and -2, initiating a negative signaling cascade that ultimately inhibits F-actin-dependent phagocytosis and immune signaling (4, 5). The phosphorylation levels of SIRPA are greatly enhanced by the engagement of the membrane protein CD47 in *trans*, which leads to the recruitment of the SHP phosphatases and the subsequent dephosphorylation of several prophagocytic proteins, including the heavy chain (MYH9) of non-muscle myosin IIA (NM-IIA) (6–8).

NM-IIA is a hexameric protein with motor and contractile properties involved in cell migration, adhesion, phagocytosis, and other cellular processes, which consists of homodimers of MYH9, regulatory light chains (RLC), and essential light chains (9). Each MYH9 monomer comprises an N-terminal motor domain, which regulates the production of mechanical force through magnesium-dependent ATP hydrolysis (ATPase activity) and binding to actin filaments, and a C-terminal cargo-binding tail domain (9, 10). Phosphorylation of the RLCs regulates the assembly of NM-IIA into bipolar filaments, which is essential to engage and contract the actin cytoskeleton (11, 12). It was shown that SIRPA limits viral endocytosis by a mechanism that resembles its inhibitory activity on phagocytosis (3), and that F-actin-mediated phagocytosis is mostly driven by phospho-activated MYH9, since its pharmacological inhibition prevents particle engulfment to a similar extent as the negative signaling initiated by the SIRPA-CD47 interaction (8, 13). Therefore, we speculated that other phagocytic proteins could also have a role in virus infection.

Here, we investigated whether MYH9 enhances viral infection and how its activity would be regulated during this process. We found that MYH9 increases infection levels of unrelated enveloped viruses, including flavivirus, arenavirus, rhabdovirus, and togavirus, which use different mechanisms of virus entry, and that its ATPase activity and self-oligomerization are essential for its proviral activity. Furthermore, we showed that virus infection drives the accumulation of MYH9 at the plasma membrane to support post-binding steps of virus entry and that phosphorylation of the tyrosine residues 277 and 1805 is critical for its function in infection. By analyzing different families of non-receptor tyrosine kinases (NRTKs), we identified that members of the Src family kinase would phosphorylate MYH9 upon viral infection, which could be therapeutically exploited.

## RESULTS

### MYH9 enhances viral infection in human and mouse cells

**Fig 1:**
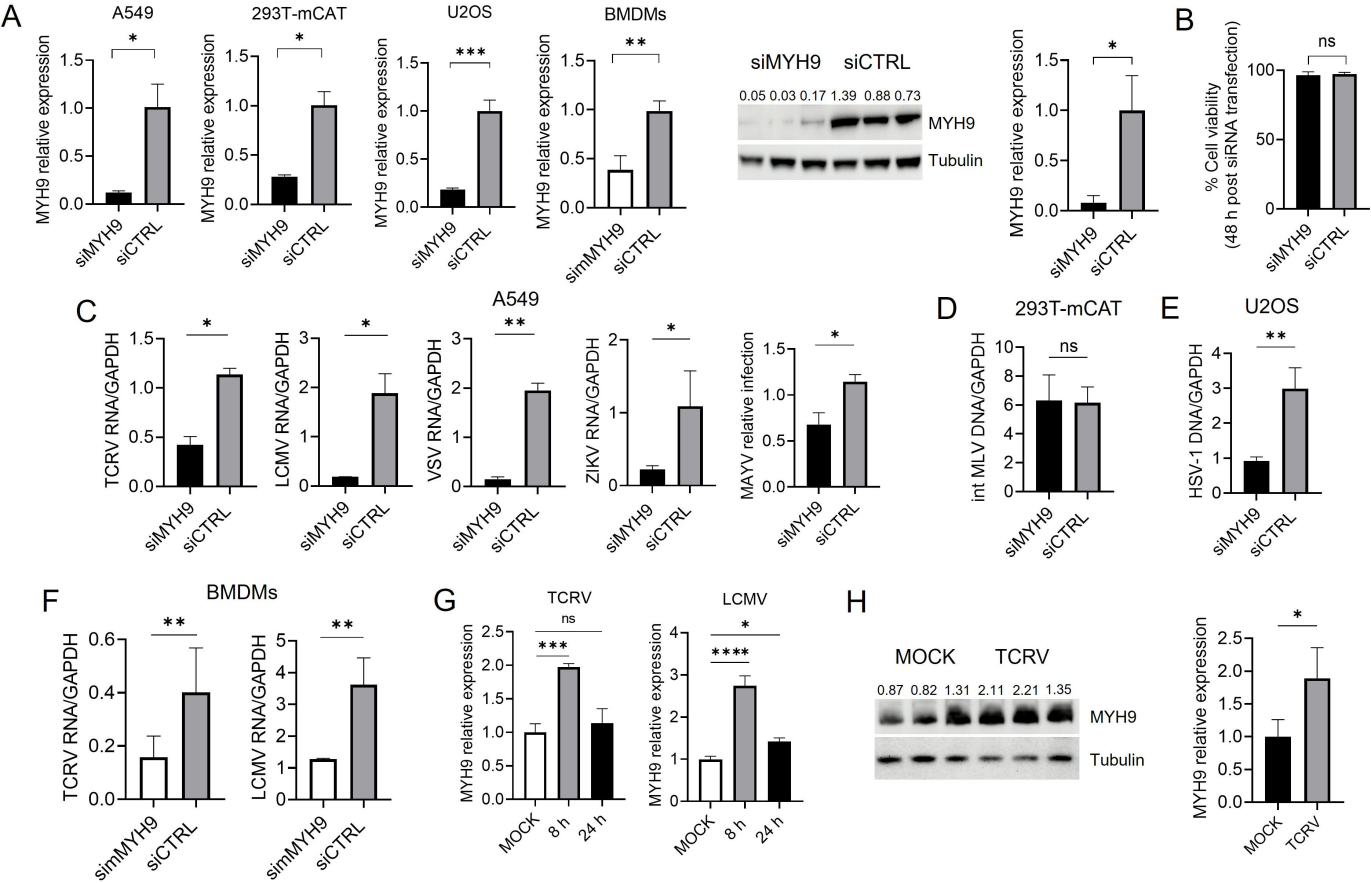
MYH9 enhances viral infection in human and mouse cells. (**A**) *MYH9* knockdown validation in human cell lines and mouse primary cells transfected with a siMYH9 or a siCTRL; *MYH9* mRNA levels were analyzed in human cell lines and mouse primary cells by RT-qPCR (left) and MYH9 expression was assessed by western blot in A549 cells using a rabbit anti-MYH9 (CST) (right) at 48 h post-siRNA transfection. Relative MYH9 expression levels from independent experiments are depicted above each lane and a mouse anti-Tubulin (Thermo) served as a control. (**B**) Analysis of cell viability in siMYH9- and siCTRL-transfected A549 cells. (**C**) A549 cells were transfected with a siMYH9 or a siCTRL as detailed, and infected with the indicated viruses for 24 h. Viral RNA levels were analyzed by RT-qPCR. (**D**) 293T-mCAT cells were transfected with the indicated siRNAs for 48 h and infected with MLV. DNA was isolated at 48 hpi and analyzed by qPCR. (**E**) HSV-1 infection levels upon MYH9 knockdown. DNA was isolated at 24 hpi and analyzed by qPCR. (**F**) BMDMs isolated from C57BL/6 mice were transfected with a mouse-specific siMYH9 and infected with TCRV and LCMV. Viral RNA was isolated 48 hpi and analyzed by RT-qPCR. Shown is the average ± standard deviation (SD) of 3–4 independent experiments. Unpaired t test was used to determine significance. ***, *P* ≤ 0.0002; **, *P* ≤ 0.009; *, *P* ≤ 0.05; ns, not significant. (**G**) A549 cells were infected with TCRV or LCMV, and endogenous *MYH9* mRNA levels were determined by RT-qPCR at 8 and 24 hpi. The data shown represent the average ± SD of three independent experiments. One-way analysis of variance (ANOVA) was used to determine significance. ****, *P* < 0.0001; *, *P* ≤ 0.03; ns, not significant. (**H**) MYH9 expression was analyzed by western blot in A549 cells infected with TCRV for 24 h using a rabbit anti-MYH9 (CST) and relative MYH9 expression levels are depicted above each lane. Shown is the average ± SD of three independent experiments. An unpaired t test was used to determine significance. *, *P* ≤ 0.04. A mouse anti-Tubulin (Thermo) was used as a control.

Given the significant role of MYH9 in distinct cellular processes that require the generation of mechanical force, *MYH9* knockout cells are mostly non-viable and *Myh9* depletion is embryonic lethal in mice (9, 14). Thus, to analyze the role of MYH9 in viral infection, we did short-interfering RNA (siRNA)-mediated knockdowns in human and mouse cells, which transiently reduced *MYH9* mRNA and protein expression (Fig. 1A), without compromising cell viability 48 h post-transfection (Fig. 1B). siMYH9-transfected cells were infected with replicative-competent viruses at a multiplicity of infection (MOI) of 1, and viral replication levels were measured at 24–48 h post-infection (hpi) by quantitative polymerase-chain reaction (qPCR). *MYH9* knockdown resulted in significantly lower infection levels of the New World arenavirus (NWA) Tacaribe (TCRV), the Old World arenavirus (OWA) Lymphocytic Choriomeningitis virus (LCMV), the flavivirus Zika (ZIKV), the rhabdovirus vesicular stomatitis virus (VSV), and the togavirus Mayaro (MAYV) (Fig. 1C), while infection levels of the retrovirus murine leukemia virus (MLV) were not reduced upon siMYH9 transfection with respect to a siRNA control (siCTRL) (Fig. 1D). As a control for this experiment, we used herpes simplex virus type-1 (HSV-1), which was first described to use MYH9 as a cellular receptor (15), observing lower HSV-1 infection levels upon *MYH9* knockdown as expected (Fig. 1E). To analyze Myh9 activity in viral infection, we isolated bone marrow-derived macrophages (BMDMs) from 8- to 12-week-old C57BL/6 mice, and we transfected a mouse-specific siRNA (simMYH9) in fully differentiated BMDMs for 48 h (Fig. 1A). Next, we infected simMYH9-transfected cells with TCRV and LCMV at an MOI = 1 for 48 h, which were shown to infect primary mouse cells at high levels (16), and we found that reduced expression of *Myh9* also resulted in a significant decrease in viral infection levels for both viruses (Fig. 1F), indicating that MYH9 proviral activity is conserved in both human and mouse cells.

We next asked whether viral infection could upmodulate the expression levels of MYH9 to further support infection. To this end, we quantified *MYH9* mRNA and endogenous protein expression in A549 cells after viral infection. We observed that *MYH9* transcript levels were increased at 8 hpi, while at 24 hpi, the expression levels were comparable to those observed for MOCK-infected cells (Fig. 1G). Western blot analysis showed that MYH9 levels were significantly increased at 24 hpi (Fig. 1H), suggesting that virus infection establishes a positive feedback-like mechanism to further increase infection levels.

### The ATPase activity and self-assembly of MYH9 promote infection of viruses exploiting different uptake mechanisms

There is now a consensus about the existence of six main endocytic pathways in mammalian cells, which can be broadly divided based on their dependence on dynamin-2 (DYN-2) activity, a large GTPase involved in pinching off endocytic vesicles from the plasma membrane (17). DYN-2-dependent endocytic processes include clathrin-mediated (CME), fast endophilin-mediated, and clathrin-independent carrier pathways, while dynamin-independent mechanisms include caveolae, macropinocytosis, and phagocytosis (17). In Fig. 1, we showed that MYH9 enhances infection of viruses that exploit different endocytic pathways to enter cells: TCRV, ZIKV, VSV, and MAYV use CME (18–21), while LCMV exploits a macropinocytosis-like (MPL) mechanism for cellular entry (22). To formally examine whether the activity of MYH9 in viral infection is independent of the virus entry route, we analyzed infection levels in *MYH9* knockdown cells upon DYN-2 inhibition; for these experiments, we used the NWA TCRV and the OWA LCMV, whose entry is DYN-2-dependent (CME) and -independent (MPL), respectively. In brief, TCRV entry is initiated by the binding of the viral glycoprotein (GP) to a yet-to-be-identified surface receptor(s). Unlike pathogenic NWAs, TCRV infects human cells independently of transferrin receptor 1 (TfR1) (2). In this context, we showed that L-type voltage-gated calcium channels are required for efficient TCRV entry (16). Subsequent to the interaction of TCRV GP with its receptors/entry factors on the cell surface, viral internalization occurs through CME, followed by trafficking to a pH 5 late endosome where virus-cell fusion occurs (2, 18). On the other hand, LCMV GP interacts with its well-characterized bona fide receptor α-dystroglycan (α-DG) on the cell surface, promoting viral internalization via a non-classic macropinocytosis mechanism where early macropinosomes deliver the virus-receptor complex to the late endosome for subsequent fusion of the viral and endolysosomal membranes facilitated by the mucin receptor CD164 (23–25).

**Fig 2:**
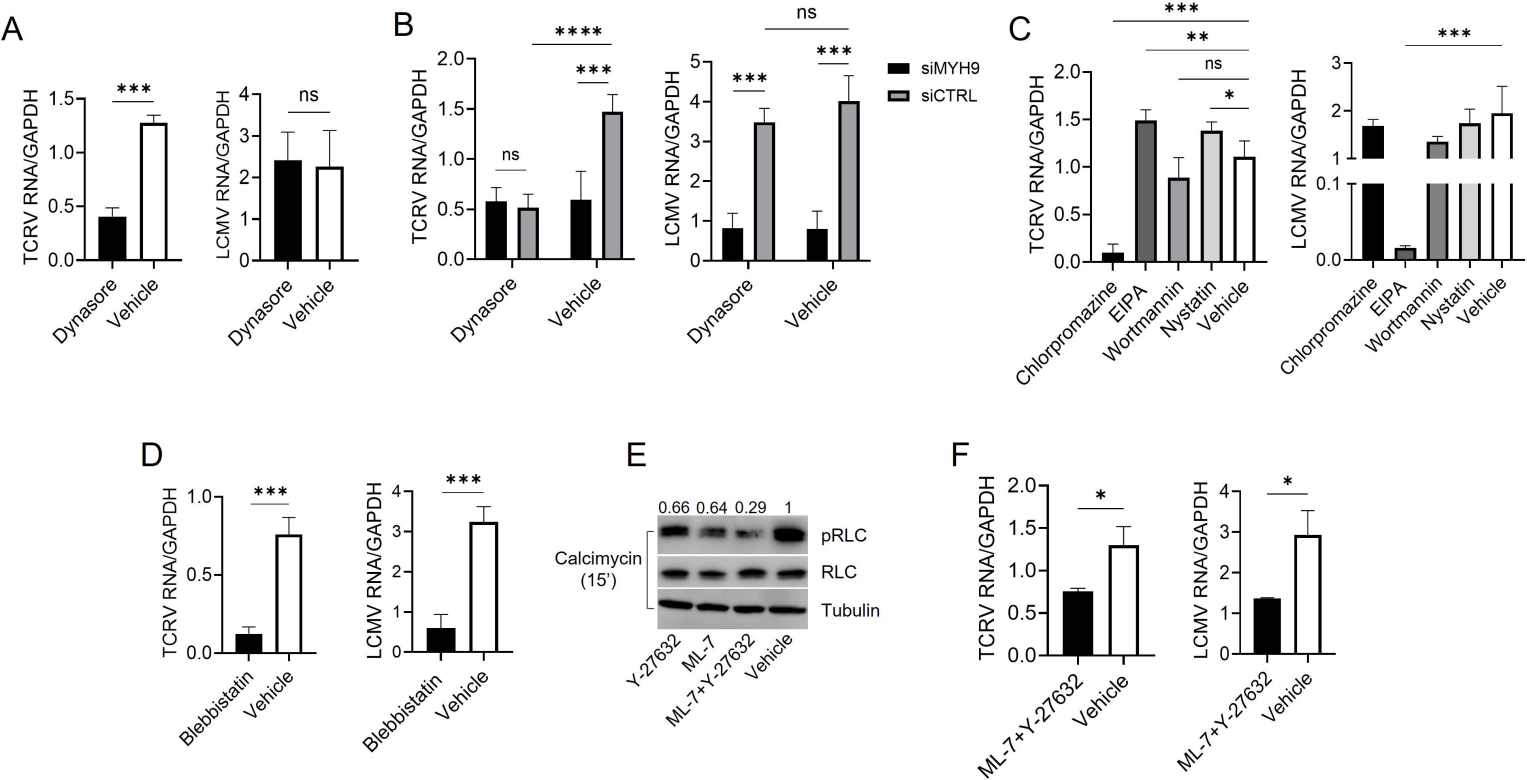
The ATPase activity and oligomerization of MYH9 promote infection of viruses exploiting different uptake mechanisms. (**A**) A549 cells were pre-treated with dynasore (100 μM) for 1 h, infected with TCRV or LCMV in the presence of the inhibitor, and the viral RNA was analyzed at 24 hpi by RT-qPCR. The data shown represent the average ± SD of four independent experiments. Unpaired t test was used to determine significance. ***, *P* ≤ 0.0001; ns, not significant. (**B**) A549 cells were transfected with a siMYH9 or a siCTRL for 48 h and treated with dynasore (100 μM) for 1 h prior to infection. TCRV and LCMV infections were analyzed as described in panel A. The data shown represent the average ± SD of three independent experiments. Two-way ANOVA was used to determine significance. ***, *P* ≤ 0.0005; **, *P* ≤ 0.005; ns, not significant. (**C**) Cells were pre-treated with the inhibitors chlorpromazine (10 μM), EIPA (35 μM), nystatin (50 μM), or wortmannin (200 nM) for 1 h, and then infected with TCRV or LCMV in the presence of the compounds. Viral RNA was analyzed at 24 hpi by RT-qPCR. The data shown represent the average ± SD of 3–4 independent experiments. One-way ANOVA was used to determine significance. ***, *P* ≤ 0.0002; **, *P* ≤ 0.004; *, *P* ≤ 0.04; ns, not significant. (**D**) A549 cells were incubated with blebbistatin (25 μM) for 1 h and then infected with TCRV or LCMV in the presence of the inhibitor for 1 h. At 24 hpi, viral RNA was isolated and analyzed by RT-qPCR. (**E**) A549 cells were incubated with ML-7 (10 μM) and/or Y-27632 (25 μM) for 45 min and then treated with calcimycin (CST) for 15 min. Total levels of RLC and pRLC (S18/T19) were determined by western blot using monoclonal antibodies from CST. Relative pRLC levels are depicted above each lane. A mouse anti-Tubulin (Thermo) was used as a control. (**F**) A549 cells were incubated with ML-7 (10 μM) and Y-27632 (25 μM) for 45 min and then infected with TCRV or LCMV for 1 h in the presence of the inhibitors. Shown is the average ± SD of three independent experiments. An unpaired t test was used to determine significance. *, *P* ≤ 0.02.

To first corroborate the role of DYN-2 in TCRV and LCMV infection, we pre-incubated A549 cells for 30 min with the inhibitor dynasore, followed by viral infections (MOI = 1) in the presence of the inhibitor for 1 h, and viral RNA levels were analyzed by reverse transcription quantitative PCR (RT-qPCR) at 24 hpi. As expected, only TCRV, but not LCMV, infection was decreased in cells treated with dynasore with respect to a vehicle control (dimethyl sulfoxide [DMSO]) (Fig. 2A). Next, we transfected a siMYH9 for 48 h before dynasore treatment and virus infection, and we analyzed TCRV and LCMV RNA levels. *MYH9* knockdown did not further decrease TCRV infection levels in dynasore-treated cells, suggesting that MYH9 enhances DYN-2-dependent TCRV infection, whereas LCMV infection levels were reduced only in those cells transfected with a siMYH9 irrespective of the treatment with dynasore (Fig. 2B), indicating that MYH9 is supporting virus infection independently of the viral entry route used. To rule out that TCRV and LCMV could use alternative entry pathways, which may be also contributing to infection and thus be targeted by the activity of MYH9, we tested the specific inhibitors chlorpromazine (CME), nystatin (caveolae), 5-(N-ethyl-N-isopropyl)amiloride (EIPA) (macropinocytosis), and wortmannin (phagocytosis) prior to and during viral infection, as described above. We found that only chlorpromazine significantly reduced TCRV infection levels, confirming that this virus mostly uses CME to enter cells (Fig. 2C). Interestingly, nystatin- and EIPA-treated cells showed increased levels of TCRV infection, which agrees with the reported upregulation of CME when other endocytic routes are perturbed/depleted (17). On the other hand, LCMV infection levels were only decreased in EIPA-treated cells due to the inhibition of MPL, its main entry route (Fig. 2C). Collectively, these data suggest that MYH9 is a pleiotropic proviral host factor that supports infection of viruses exploiting both DYN-2-dependent and -independent uptake mechanisms.

Many of the cellular functions performed by NM-IIA require the generation of motor/contractile force by the head domain of MYH9, which may be suppressed either by inhibiting its Mg^2+^-dependent ATPase activity or by preventing the serine/threonine phosphorylation of the RLCs (9–12). Thus, to determine the molecular basis of the activity of MYH9 in viral infection, we first used the inhibitor blebbistatin, which binds to MYH9 ATPase site, hence suppressing the overall mechanical activity of NM-IIA (26). The treatment of A549 cells with blebbistatin before and during infection resulted in a significant reduction in TCRV and LCMV RNA levels at 24 hpi, suggesting that the ATPase activity is necessary for the proviral function of MYH9 (Fig. 2D).

It was also shown that phosphorylation of threonine (T) 18 and serine (S) 19 residues in the RLCs of NM-IIA by myosin light chain kinase (MLCK) and Rho-associated protein kinases (ROCK) regulates the assembly of bipolar filaments that engage and contract actin filaments (11, 12). Interestingly, these kinases are simultaneously active and compete for a limiting pool of cellular NM-IIA monomers; thus, the assembly of NM-IIA filaments could be driven not only by direct phosphorylation/activation of the T18/S19 residues by a given kinase, but also by inactivating the competing kinase(s) (27, 28). To first corroborate that the phosphorylation of the RLC would not be completely prevented by only inhibiting MLCK or ROCK kinases, we analyzed the phosphorylation of the T18/S19 residues by western blot, upon treatment with the inhibitors ML-7 (MLCK) and Y-27632 (ROCK) in A549 cells pre-incubated with the calcium ionophore calcimycin to promote the phosphorylation of the RLCs. The individual treatment with ML-7 (10 μM) or Y-27632 (25 μM) modestly decreased phosphorylation of T18/S19; however, phosphorylation of the RLCs upon calcimycin treatment was markedly reduced when both inhibitors were used simultaneously (Fig. 2E). To test whether inhibiting the phosphorylation of RLCs modulates the levels of viral infection, we infected A549 cells with LCMV or TCRV in the presence of both ML-7 and Y-27632. Viral infection was analyzed 24 hpi, detecting a significant decrease in infection levels in those cells treated with the inhibitors with respect to control cells (Fig. 2F). Hence, preventing the assembly of NM-IIA in bipolar filaments, which is essential for the motor/contractile activity of MYH9, also reduces viral infection.

### Viral invasion drives the translocation of MYH9 to the plasma membrane

**Fig 3:**
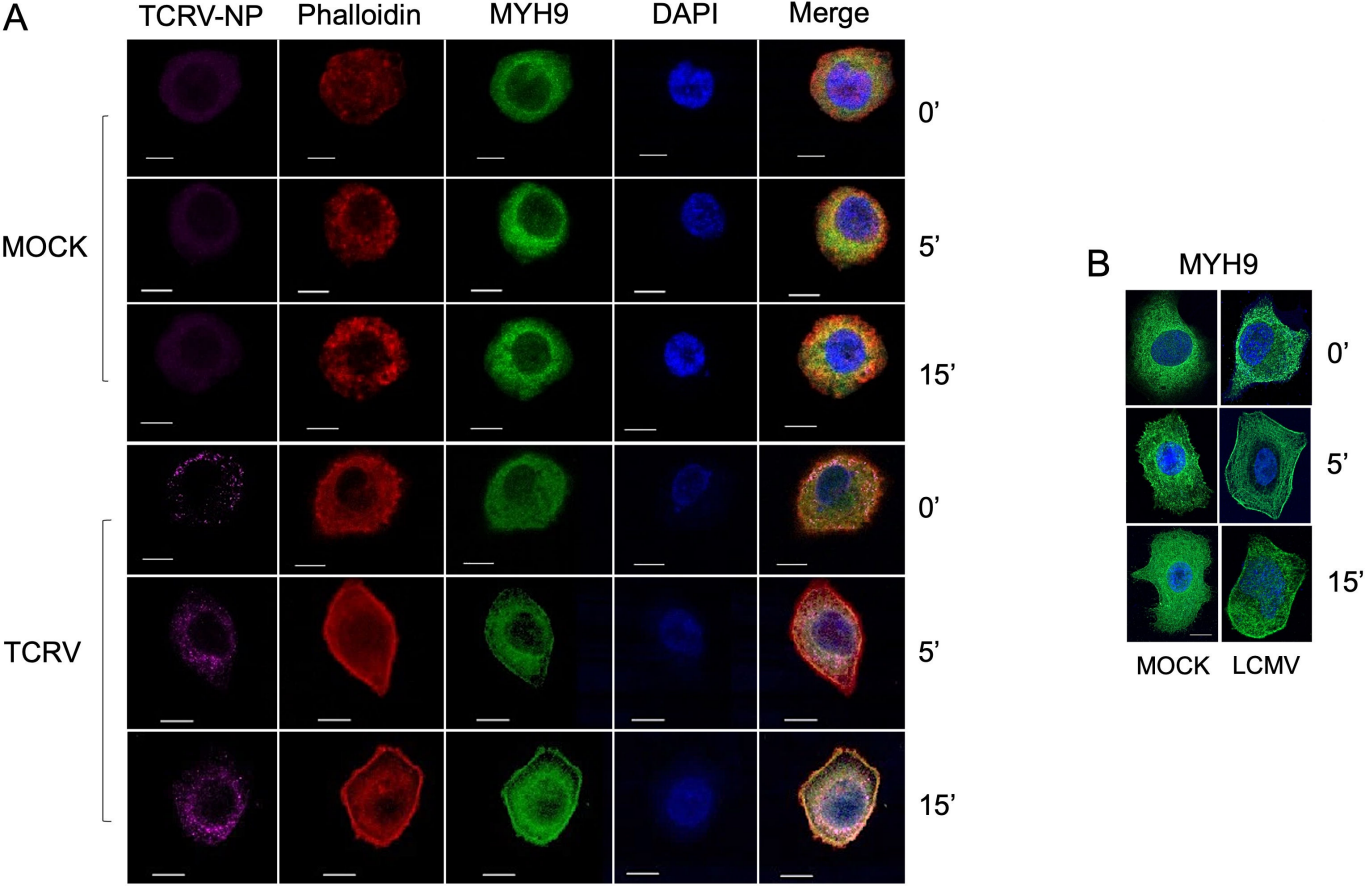
Viral infection drives the translocation of MYH9 to the cell periphery during virus entry. (**A**) A549 cells were infected with TCRV (MOI = 50) or MOCK-infected and MYH9 expression was analyzed by confocal microscopy using a rabbit anti-MYH9 (Sigma) after virus-cell binding on ice (0′) and during viral internalization at 37°C at different timepoints (5′ and 15′). TCRV nucleoprotein (TCRV-NP) was detected using a cross-reactive anti-Junín virus antibody (BEI resources), actin filaments were stained with Phalloidin-Texas Red (Invitrogen), and DAPI (VectorLabs) was used as a nuclear counterstain. Scale bar: 10 μm. (**B**) A549 cells were infected with LCMV (MOI = 50) or MOCK-infected, and MYH9 expression was analyzed during virus-cell binding and viral internalization as described in panel **A**. Scale bar: 10 μm.

Phagocytosis is a finely coordinated process that involves the assembly of the cytoskeleton and the rapid accumulation of prophagocytic proteins at the phagocytic synapse to drive engulfment, including F-actin, NM-IIA, paxillin, and actinin (29). Given the fundamental function of MYH9 during phagocytic uptake and its role as a surface receptor for HSV-1 (8, 15), we next asked whether MYH9 might function during entry of the viruses herein analyzed. To this end, we assessed the sub-cellular localization of endogenously expressed MYH9 during viral entry by confocal microscopy. Specifically, we analyzed the expression pattern of MYH9 after TCRV binding (MOI = 50) on ice for 1 h (0′) and at different intervals during viral internalization at 37°C (5′ and 15′), using a monoclonal anti-MYH9 and a cross-reactive anti-Junín virus antibody that binds to TCRV nucleoprotein (NP). We found that virus binding to A549 cells did not alter the localization of MYH9 with respect to MOCK-infected cells, as it was uniformly distributed along the cytoplasm (Fig. 3A; MOCK 0′, 5′, 15′ and TCRV 0′). However, when TCRV-infected cells were shifted to 37°C, a permissive temperature for viral internalization, we observed a marked accumulation of MYH9 at the plasma membrane in a time-dependent fashion, which was not observed in MOCK cells (Fig. 3A; TCRV 5′, 15′). Since other cytoskeletal proteins also accumulate at the plasma membrane during phagocytosis and viral endocytosis, we also analyzed whether actin would translocate to the membrane by staining for actin filaments with Phalloidin-Alexa Fluor 568. We found that actin expression was also enriched at the cell periphery and that it increasingly colocalized with MYH9 during TCRV internalization at 37°C, which again was not observed for MOCK-infected cells (Fig. 3A), suggesting that these motor proteins collectively support TCRV endocytosis. Although we could not stain the NP of LCMV particles using a commercial antibody (clone VL4), we did observe the translocation of MYH9 during LCMV internalization, but not upon binding of viral particles (Fig. 3B; LCMV 5′, 15′). Thus, MYH9 may facilitate the internalization step of TCRV and LCMV entry when it translocates to the cell periphery.

### MYH9 supports post-binding steps of virus entry

**Fig 4:**
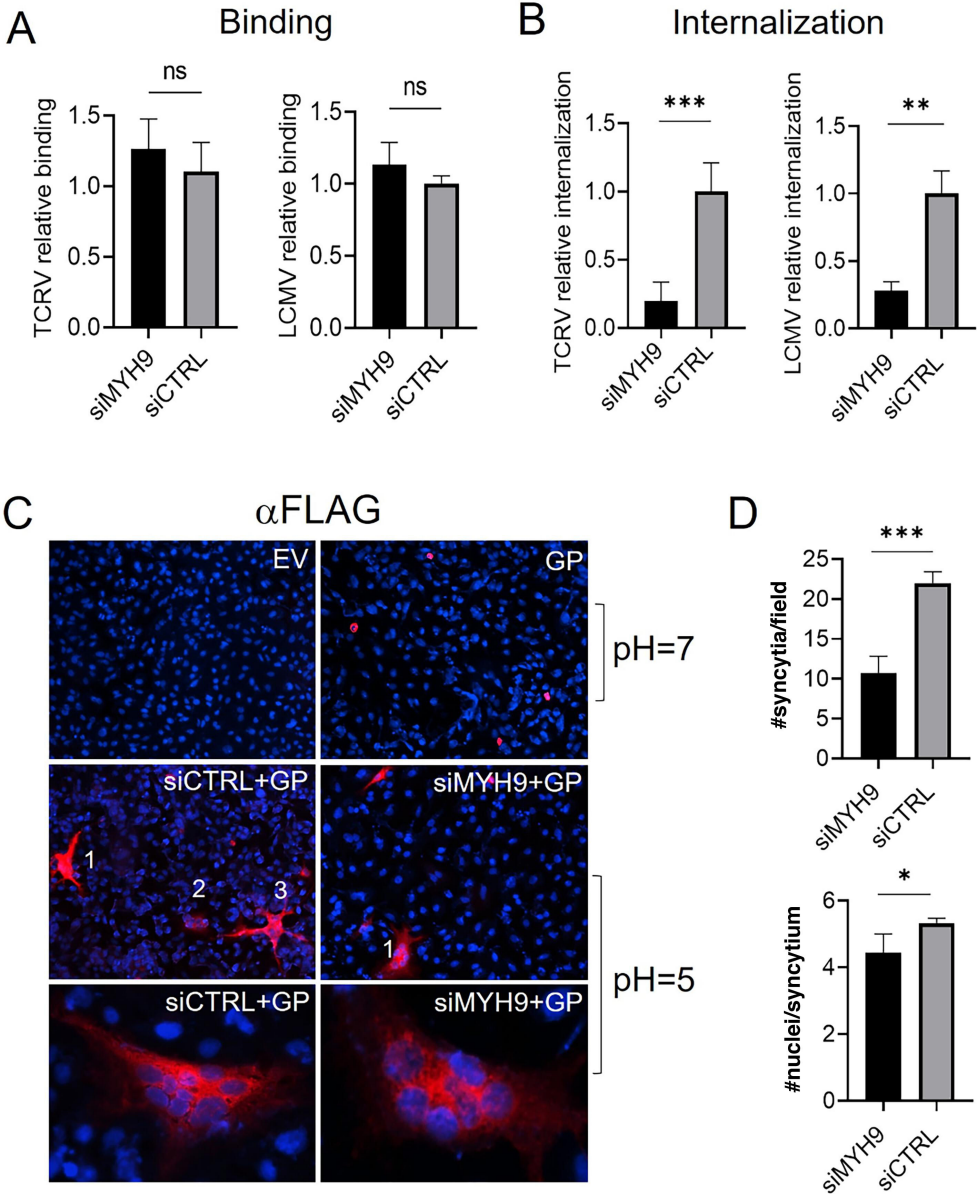
MYH9 activity supports post-binding steps of virus entry. (**A**) A549 cells were incubated with TCRV or LCMV (MOI = 10) for 1 h on ice, and RNA levels of bound virions were analyzed by RT-qPCR. Shown is the average ± SD of three independent experiments, and an unpaired t test was used to determine significance; ns, not significant. (**B**) A549 cells were infected on ice as described above, shifted to 37°C for 45 min, treated with 1 mg/mL of Proteinase K (Gibco) for 45 min, followed by 2 mM of phenylmethylsulfonyl fluoride (PMSF) (Sigma), and internalized viral RNA was quantified by RT-qPCR. Shown is the average ± SD of three independent experiments, and an unpaired t test was used to determine significance. ***, *P* ≤ 0.0008; **, *P* ≤ 0.003. (**C**) A549 cells were co-transfected with a siMYH9 and a FLAG-tagged GP for 48 h, and transfected cells were then pulsed with sodium citrate at pH = 5 or pH = 7 for 10 min to analyze syncytia formation. (**D**) The number of syncytia (top) and the number of cells per syncytium (bottom) were quantified. Shown is the average ± SD of three independent experiments, and four randomly selected regions were analyzed in each experiment. Unpaired t test was used to determine significance. ***, *P* ≤ 0.0001; *, *P* ≤ 0.03.

To formally determine which step(s) of virus entry is promoted by the activity of MYH9, we next performed virus binding, internalization, and fusion assays with variable expression levels of MYH9 in A549 cells. In brief, TCRV and LCMV binding were assessed by quantifying viral RNA from bound particles to siMYH9- and siCTRL-transfected cells after 1 h on ice, while internalized viral RNA was analyzed after virus binding, incubation of infected cells at 37°C for 45 min and stripping off non-internalized viral particles with Proteinase K. *MYH9* knockdown did not alter TCRV nor LCMV binding to cells but significantly decreased internalization levels of both viruses (Fig. 4A and B). Lastly, to determine whether MHY9 would modulate virus-cell fusion, we analyzed syncytium formation as a surrogate assay. To this end, A549 cells were co-transfected with a siMYH9 and a FLAG-tagged LCMV GP construct, and 48 h later, the transfected cells were pulsed with sodium citrate (pH = 5 or pH = 7) to assess the number and size of cell syncytia by immunofluorescence. As depicted in Fig. 4C, there was no syncytium formation when GP-transfected cells were pulsed at pH = 7, given the requirement of an acidic environment for arenavirus GP-mediated cell fusion (30). When the cells were pulsed at pH = 5, we observed a significantly lower number of syncytia, as well as cells per syncytium in siMYH9-transfected cells, with respect to siCTRL-transfected cells (Fig. 4C and D), indicating that MYH9 is also required for an efficient process of virus-cell fusion.

### Tyrosine phosphorylation of MYH9 is essential for its role in virus entry

**Fig 5:**
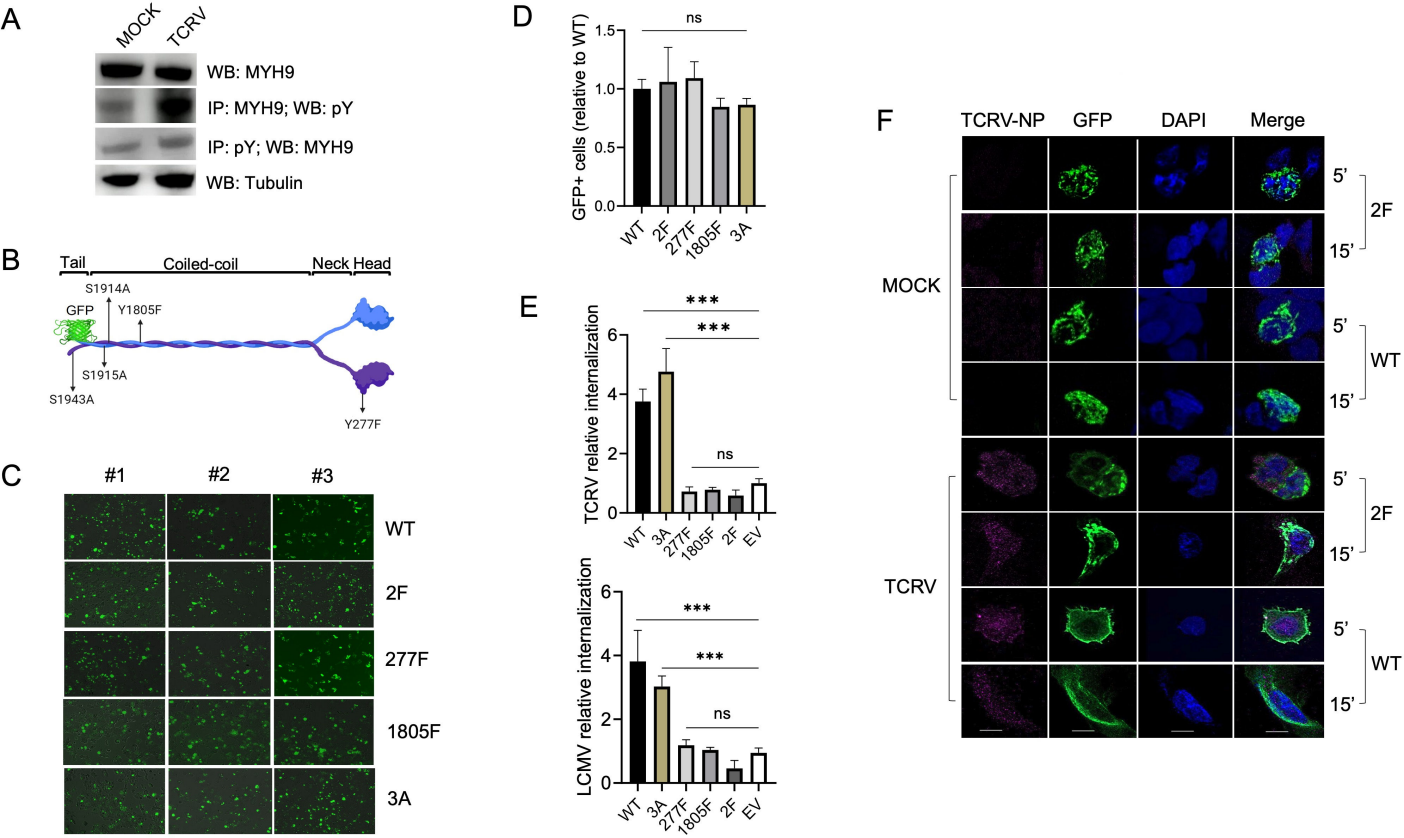
Tyrosine phosphorylation of MYH9 is essential for its function in virus entry. (**A**) A549 cells were infected with TCRV (MOI = 10) for 15 min, and tyrosine phosphorylated (pY) proteins or MYH9 were immunoprecipitated with a mix of rabbit anti-pY antibodies (CST) or a mouse anti-MYH9 (Sigma), respectively. MYH9 expression (total and pY) was analyzed by western blot with a rabbit anti-MYH9 (CST). A mouse anti-Tubulin (Thermo) was used as a control. (**B**) Graphic scheme of MYH9 fused to green fluorescent protein (GFP), showing the protein domains and the localization of the substituted residues in the mutant constructs (created with BioRender). (**C**) MYH9 WT and mutant constructs were overexpressed in A549 cells for 24 h and GFP expression was assessed by epifluorescence microscopy in three independent experiments. (**D**) Quantification of GFP+ cells 24 h post-transfection of the MYH9 constructs. One-way ANOVA was used to determine significance; ns, not significant. (**E**) TCRV (top) and LCMV (bottom) internalization assays in A549 cells overexpressing MYH9 WT and mutant constructs. Shown is the average ± SD of three independent experiments. One-way ANOVA was used to determine significance. ***, *P* ≤ 0.0001; ns, not significant. (**F**) A549 cells were transfected with the MYH9 WT and 2F constructs for 24 h and infected with TCRV (MOI = 50). GFP and TCRV-NP expression was determined by confocal microscopy during viral internalization at 37°C (5′ and 15′). DAPI (VectorLabs) was used as a nuclear counterstain. Scale bar: 10 μm.

Although MYH9 participates in several cellular processes requiring the generation of mechanical/contractile force, to our knowledge, there are a few studies analyzing how its activity is regulated by tyrosine phosphorylation (pY). Of note, MYH9 is phospho-activated in different tyrosine residues upon B cell antigen receptor (BCR) stimulation, bacterial invasion, and to efficiently drive F-actin-driven phagocytosis (8, 31, 32). To analyze whether virus invasion triggers pY of MYH9, we infected A549 cells with TCRV for 15 min, and we either immunoprecipitated tyrosine-phosphorylated proteins using a mix of anti-pY antibodies or MYH9 with a monoclonal antibody. Western blot analysis showed that pY levels of MYH9 were significantly increased in TCRV-infected cells with respect to MOCK-infected control cells (Fig. 5A), indicating that virus entry triggers pY and activation of MYH9.

Tsai et al. demonstrated that pY of 277 and 1805 residues is essential for the function of MYH9 in phagocytic engulfment, given that point mutations in any of these residues inhibited its contractile function during the process (8). To analyze whether pY of these key residues would also modulate the function of MYH9 in virus entry, we mutated 277Y and 1805Y to phenylalanine (F) individually or in combination (227F, 1805F, and 277,1805F [2F]), using a construct of MYH9 WT fused to green fluorescent protein (GFP) as template (Fig. 5B). We also analyzed the MYH9 3A mutant, which harbors alanine (A) replacements in three serine (S) residues (1914S, 1915S, and 1946S), whose phosphorylation is essential for the activity of MYH9 in focal adhesion and protrusion extension (Fig. 5B) (33). To examine the contribution of these mutant versions of MYH9 to virus entry, we first assessed and quantified the expression of GFP in transfected cells, finding that the MYH9 227F, 1805F, 2F, and 3A mutants expressed at similar levels with respect to the MYH9 WT construct (Fig. 5C and D). Interestingly, forced expression of MYH9 WT resulted in a gain-of-function in viral internalization, while we did not observe an increase in internalization levels in 227F-, 1805F-, or 2F-expressing cells, with respect to an empty vector (EV) control (Fig. 5E). Overexpression of the 3A mutant enhanced viral internalization to similar levels to the WT construct (Fig. 5E), indicating that substituting 277Y and 1805Y residues, but not 1914S, 1915S, and 1946S, abrogates the activity of MHY9 during virus entry.

Since we found that the MYH9 phospho-inactive mutants cannot support virus entry although their expression levels were comparable to the WT construct, we then asked whether these mutants may have an impairment to translocate to the cell periphery to promote viral entry. To test this, we analyzed GFP expression in MYH9 WT- and 2F-transfected cells during TCRV internalization, an entry step enhanced by the activity of MYH9, by confocal microscopy. In MOCK-infected cells, both MYH9 WT and 2F constructs were distributed in the cytoplasm when incubated at 37°C for 5′ or 15′ (Fig. 5F; MOCK 5′, 15′). However, the MYH9 2F mutant did not accumulate at the plasma membrane during viral internalization, as it was observed for the WT protein for both time points (Fig. 5F; TCRV 5′, 15′). Collectively, these results indicate that pY of the 277 and 1805 residues is required for the translocation of MYH9 to the cell periphery, and hence for its proviral activity during viral entry.

### Src family kinases likely phosphorylate MYH9 upon virus entry

**Fig 6:**
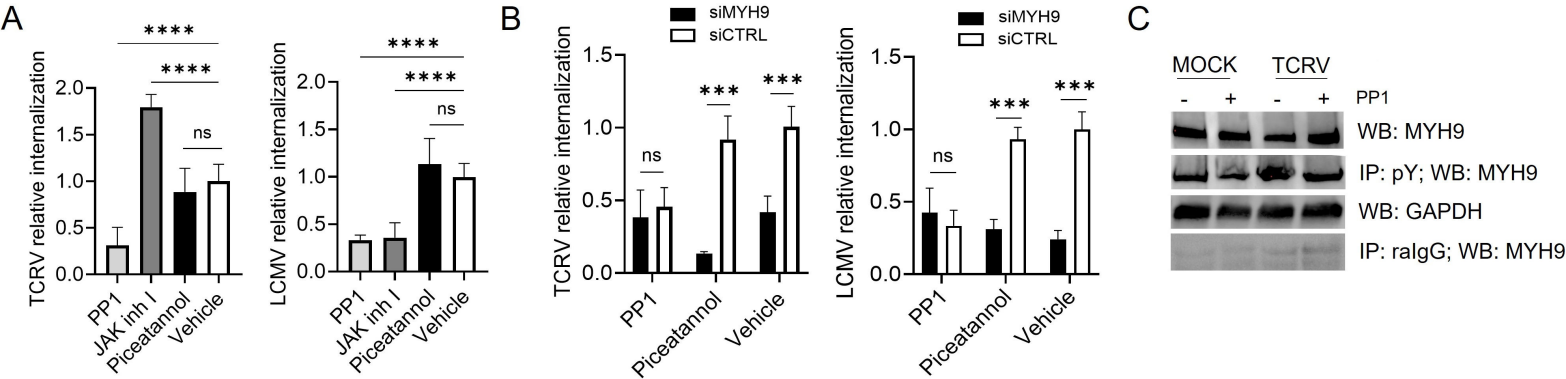
Src family kinases likely phosphorylate MYH9 upon virus entry. (**A**) A549 cells were pre-treated with the NRTK inhibitors PP1 (50 μM), Jak inhibitor I (100 nM), or piceatannol (50 μM) for 45 min, and viral internalization was analyzed in the presence of the inhibitors. Shown is the average ± SD of three independent experiments. One-way ANOVA was used to determine significance. ****, *P* < 0.0001; ns, not significant. (**B**) A549 cells were transfected with a siMYH9 or a siCTRL for 48 h, treated with the indicated inhibitors for 45 min prior to infection, and viral internalization was analyzed in the presence of the inhibitors. Two-way ANOVA was used to determine significance. ***, *P* ≤ 0.0003; **, *P* ≤ 0.001; ns, not significant. (**C**) A549 cells were pre-treated with PP1 (50 μM) or DMSO (vehicle) for 45 min, infected with TCRV (MOI = 10) or MOCK-infected for 15 min, and tyrosine phosphorylated proteins were immunoprecipitated with a mix of rabbit anti-pY antibodies (CST). MYH9 expression (total and pY) was analyzed by western blot with a rabbit anti-MYH9 (CST). Immunoprecipitation with a rabbit IgG polyclonal antibody and detection of GAPDH served as controls.

To further characterize the role of pY in infection, we lastly sought to identify which kinase(s) phosphorylate MYH9 upon virus entry. There are nine families of NRTKs in mammals, known as Src, Abl, Fes, Jak, Ack, Syk, Tec, Fak, and Csk (34), which could phosphorylate/activate MYH9 to promote infection. To analyze this, we investigated whether the activity of Src, Jak, and/or Syk kinases would modulate virus entry by using selective inhibitors. Briefly, A549 cells were pre-treated with small molecules targeting Src (PP1), Syk (piceatannol), and Jak (Jak inhibitor I) families for 45 min, to later assess viral internalization in the presence of the inhibitors. PP1 treatment markedly reduced internalization levels of both viruses, piceatannol did not affect TCRV nor LCMV internalization, while Jak inhibitor I had opposite effects: it greatly enhanced TCRV internalization but decreased the process for LCMV (Fig. 6A), indicating that members of the Src family phosphorylate cellular proteins relevant for both TCRV and LCMV entry.

Since Src kinases phosphorylate hundreds of proteins to modulate their function in several physiological and pathological processes (35), we coupled *MYH9* knockdown with PP1 treatment to analyze whether these kinases may be activating MYH9 upon virus infection. Interestingly, TCRV and LCMV internalization levels were similarly decreased in siMYH9- and siCTRL-transfected cells pre-incubated with PP1, while piceatannol-treated cells showed decreased internalization levels only when transfected with a siMYH9 but not with a siCTRL, as was observed for the vehicle (DMSO) control (Fig. 6B). These results suggest that members of the Src family may phosphorylate MYH9 during virus entry, since PP1 treatment did not further reduce viral internalization levels in siMYH9-transfected cells. To further analyze this, we immunoprecipitated pY upon TCRV infection (15 min) in PP1-treated and control cells, as well as in MOCK-infected cells. Western blot analysis showed that pY of MYH9 during TCRV invasion was diminished in the presence of PP1 to levels comparable to those detected in MOCK cells (Fig. 6C); as expected, we did not detect MYH9 when using an IgG control antibody (raIgG) for the immunoprecipitation (Fig. 6C). These results support that Src kinases are likely to phosphorylate and activate MYH9 during virus entry to further promote infection.

## DISCUSSION

NM-IIA is a ubiquitous actin-binding protein with critical functions in a wide range of cellular physiological processes, including cell adhesion, migration, and phagocytosis, and more recently it has also been implicated in modulating viral and bacterial infection (9, 36). NM-IIA is a molecular motor that provides mechanical force through ATP hydrolysis; hence, its function in infection is often associated with the motor and contractile properties of MYH9 (36). MYH9 was first identified as a cellular receptor for HSV-1, and later studies showed that it also promotes infection of other herpesviruses (Kaposi’s-sarcoma associated Herpesvirus; KSHV) and arterivirus (severe fever with thrombocytopenia syndrome virus and porcine reproductive and respiratory syndrome virus) (15, 37–40). Here, we showed that MYH9 enhances infection of endocytic viruses from divergent families, including *Arenaviridae, Flaviviridae, Rhabdoviridae,* and *Togaviridae*, and that such activity is conserved in human and mouse cells (Fig. 1C and F). We also found that viral infection upregulates MYH9 endogenous expression, suggesting a virus-driven positive regulatory mechanism (Fig. 1G and H). This observation is not without precedent, since the upregulation and/or activation of cellular proteins to support infection has been reported. For instance, severe acute respiratory syndrome coronavirus 2 (SARS-CoV-2) infection upregulates the expression of the viral receptor ACE2 through the transcription factor GATA6, while Influenza A virus infection enhances galectin-3 expression to modulate the activity of the viral polymerase complex (41, 42). Thus, MYH9 is a broad proviral host factor whose expression is positively modulated to increase viral infection levels by a mechanism currently under investigation.

By analyzing TCRV and LCMV infection, which, respectively, use CME and MPL for cell entry, we showed that MYH9 supports infection of viruses exploiting both dynamin-dependent and -independent uptake processes (Fig. 2B and C). Interestingly, it was shown that NM-IIA supports entry of KSHV by CME in foreskin fibroblasts and by macropinocytosis in dermal endothelial cells, which are both dependent on the activation of the E3 ubiquitin ligase c-Cbl by tyrosine phosphorylation (37, 38). KHSV interacts with and activates Ephrin A2 early during infection, which in turn associates with phospho-c-Cbl, NM-IIA, as well as clathrin and its adapter protein AP2 to promote CME (37). In the case of macropinocytosis, the interaction of phospho-c-Cbl with phospho-RLC in KSHV-induced blebs leads to the association of NM-IIA with actin filaments to accelerate actomyosin contraction and bleb retraction (38). Future studies will be aimed at determining whether MYH9 forms part of a conserved protein signaling complex to promote distinct viral endocytic routes.

To get further insight into the proviral activity of MYH9, we sought to analyze the effect in viral infection of decreasing the motor/contractile activity of NM-IIA or preventing its self-assembly into bipolar filaments. The small molecule blebbistatin binds to the ATPase site of MYH9, decreasing the phosphate release rate and thereby suppressing its motor activity, which is needed for efficient phagocytic engulfment (8, 26, 43). In blebbistatin-treated cells, TCRV and LCMV infection levels were greatly reduced (Fig. 2D). Moreover, we determined that preventing the phosphorylation of the RLCs in the S18/T19 residues, which modulates the self-assembly of NM-IIA monomers into functional bipolar filaments that interact with the actin cytoskeleton (11, 28), also reduced virus infection (Fig. 2E and F). These results hence indicate that the motor/contractile activity and the self-oligomerization of NM-IIA are essential for the proviral function of MYH9.

Although we showed that MYH9 is a host factor that enhances infection of divergent endocytic viruses, its role in viral infection was first described for HSV-1; Arii et al. proposed that MYH9 functions as a cellular receptor for HSV-1, favoring virus binding and virus-cell fusion at the plasma membrane by directly interacting with the viral gB glycoprotein (15). Later studies suggested that MYH9 would also promote cell binding of arteriviruses by interacting with surface glycoproteins (39, 40), although it was not established how MYH9, a cytoplasmic protein, would become inserted or expressed at the cell surface to promote virus binding (44). We examined whether MYH9 may serve as an entry factor for TCRV and LCMV by analyzing its sub-cellular localization upon infection. We found that virus binding did not induce the accumulation of MYH9 at the cell periphery, and that the levels of viral RNA from bound particles were similar in siMYH9- and siCTRL-transfected cells (Fig. 3A, B, and 4A). However, when the infected cells were shifted to a permissive temperature for virus internalization, we observed a sustained increase in the expression of membrane-associated MYH9 and a marked decrease in internalized viral RNA in *MYH9* knockdown cells (Fig. 3A, B, and 4A), indicating that MYH9 expression is relevant for virus uptake but not for virus-cell binding. We also detected actin enrichment and increased co-localization with MYH9 during viral internalization, suggesting that these motor proteins cooperate to enhance this process (Fig. 3A). Lastly, we used a surrogate system to determine whether MYH9 would also modulate virus-cell fusion. Arenavirus glycoproteins are class I fusion proteins that get primed at an acidic pH to mediate viral-endosomal membrane fusion (45). Our fusion assay showed that *MYH9* knockdown significantly reduced the formation of cell syncytia and the number of cells per syncytium at pH = 5 (Fig. 4C and D), indicating that MYH9 is likewise important for virus-cell fusion. Collectively, our results demonstrate that MYH9 translocates to the plasma membrane upon infection to support post-binding steps of viral endocytosis.

We next investigated how the function of MYH9 in virus entry may be regulated, because it was not previously addressed (15, 39, 40). In fact, little is known about how post-translational modifications in MYH9 modulate the activity of NM-IIA in other cellular processes that require motor/contractile force (9, 10). To our knowledge, there are three studies analyzing the role of tyrosine phosphorylation of MYH9 in phagocytosis, BCR stimulation, and bacterial invasion (8, 31, 32). Specifically, phosphorylation of the Y277/1805 residues favors the polymerization of NM-IIA dimers into bipolar filaments, which is essential for phagocytic engulfment, while *Listeria monocytogenes* infection triggers the phosphorylation of Y158 which affects the ability of MYH9 to bind to and/or hydrolyze ATP thus preventing bacterial dissemination (8, 32). Upon demonstrating that viral invasion triggers tyrosine phosphorylation of MYH9 (Fig. 5A), we examined whether phosphorylation of Y277 and/or Y1805 might regulate its function in virus infection. We first analyzed these residues because we showed that the contractile activity of MYH9 is needed to enhance entry (Fig. 2D), and we hypothesize that phagocytic proteins would also have a role in viral endocytosis. We showed that the MYH9 phospho-inactive mutants 277F, 1805F and 2F cannot functionally contribute to virus entry, indicating that phosphorylation of both tyrosine residues is needed for the proviral activity of MYH9 (Fig. 5E). Furthermore, we also determined that MYH9 2F had an impairment to translocate to and accumulate at the plasma membrane to facilitate entry (Fig. 5F). Thus, our results indicate that viral invasion triggers tyrosine phosphorylation of MYH9, which is essential to regulate its contractile activity to promote virus entry.

Lastly, we sought to determine which tyrosine kinase(s) would phosphorylate/activate MYH9 to enhance virus entry. Typically, viral infection involves the activation of many surface molecules and downstream proteins/signaling pathways, including receptor and non-receptor kinases, cytoskeletal proteins, among others (1). In this regard, it was shown that binding of the OWA Lassa virus GP to its bona fide receptor α-DG induces its tyrosine phosphorylation by NRTKs, which regulates the internalization of the virus-receptor complex (46). Here, we examined whether the Jak, Syk, and/or Src families of NRTKs may phosphorylate MYH9 upon virus infection. We initially analyzed these NRTKs because of their role in endocytic processes and virus entry: Syk kinases phosphorylate C-type lectin receptors which are used as entry factors (47, 48), Jak kinases modulate CME (49), and Src kinases phosphorylate different proteins to facilitate virus entry and replication (50–52). Moreover, members of the Src family phosphorylate the Y158 residue of MYH9 during bacterial invasion (32). We found that inhibiting the Src kinases with the small molecule PP1 decreased internalization of both TCRV and LCMV (Fig. 6A). Thus, we next analyzed the effect of combining PP1 treatment and *MYH9* knockdown to examine whether Src kinases may phosphorylate MYH9 upon virus entry. PP1 treatment did not further decrease viral internalization in siMYH9-transfected cells with respect to control cells, and the addition of the inhibitor reduced virus-triggered tyrosine phosphorylation of MYH9 during entry (Fig. 6B and C), suggesting that MYH9 may be activated by Src kinases upon infection. However, these observations need to be further corroborated by using selective inhibitors of individual members of the Src family, *Src* knockout systems, and by performing *in vitro* kinase assays. Since MYH9 expresses at high levels in many cell types and it is involved in several cellular processes, it cannot be depleted nor inhibited for therapeutic purposes (9). Thus, identifying the kinase(s) that activate MYH9 upon virus entry may provide new druggable targets to limit the early steps of infection of a variety of RNA viruses of public health importance.

## MATERIALS AND METHODS

### Mice

C57BL/6 mice were housed according to the policies of the Animal Care Committee of the Institut Pasteur de Montevideo. BMDMs were isolated from the hind limbs of 8- to 12-week-old mice as previously described (3).

### Cell lines and viruses

Vero, A549, U2OS, BHK-21, NIH-3T3, HeLa, and 293T cells were grown in Dulbecco’s modified Eagle Medium (DMEM; Gibco) supplemented with 2 mM glutamine, 10% fetal bovine serum (FBS, Invitrogen) and penicillin (100 U/mL)–streptomycin (100 µg/mL) (Invitrogen) (DMEM complete). 293T cells stably expressing mouse mCAT-1 (293T-mCAT) were also cultured with geneticin (1 µg/mL). TCRV, VSV, ZIKV (PRVABC59 strain), and HSV-1 (KOS strain) were propagated in Vero cells, while LCMV (Armstrong strain) was grown in BHK-21 cells. Briefly, cell monolayers were infected at 70%–80% confluence at an MOI of 0.01–0.03, culture medium was removed at 24 hpi, and the cells were washed with phosphate-buffered saline (1× PBS) and refed with DMEM containing 2% FBS. Culture supernatant from TCRV, VSV, ZIKV, and LCMV infected cells was collected at 4–8 dpi, passed through a 0.45 µm filter, concentrated with Vivaflow 50 system filters (Sartorius), and stored at −80°C until use. For HSV-1, cells were cultured for 2–3 dpi until 100% of cells displayed cytopathic effect (CPE). Cells were then frozen at −80°C and thawed at 37°C three times, and the cell suspension was sonicated three times for 30 s to release virus particles. Moloney MLV was harvested from the supernatants of stably infected NIH 3T3 fibroblasts. The supernatant was passed through a 0.45 µm filter and treated with 20 U/mL DNase-1 (Roche) at 37°C for 30 min. MAYV was obtained by transfecting a cDNA infectious clone (53) in HeLa cells using Lipofectamine 3000 (Thermo). Infectious MAYV particles were collected from culture supernatant when full CPE was observed.

### Virus titration

TCRV titers were determined by an infectious center assay using a mouse monoclonal anti-Junín nucleoprotein (clone IC06-BE10, BEI resources) and an anti-mouse IgG-Alexa Fluor 488 secondary antibody (Invitrogen). LCMV, ZIKV, VSV, and HSV-1 titers were established by plaque assays in Vero cells using 1% agarose overlays. MLV titers were determined by a focus-forming assay on NIH-3T3 cells by using a monoclonal anti-ENV (Ab538), as previously described (3).

### RNA interference

Silencer Select siRNAs were purchased from Thermo. Human (#4390824; ID: s534066) and mouse (#4390771; ID: s70267) MYH9 siRNAs and a negative control (siCTRL) were used following a forward-transfection protocol with Lipofectamine RNAiMax (Invitrogen). Briefly, adherent cells were transfected at 60%–70% confluence with the indicated siRNAs for 48 h, and knockdowns were verified by RT-qPCR using the primers described in Table 1. siRNA-transfected cells were then infected for 24–48 h or used for virus entry assays.

**TABLE 1:** Primer pairs used for RT-qPCR

Primer name	Primer sequence
LCMV-F	AGA ATC CAG GTG GTT ATT GCC
LCMV-R	GTT GTA GTC AAT TAG TCG CAG C
VSV-F	TGA ATG TGC CTC GTT CAG ATA
VSV-R	CCA AAG TCG ATC AAA TAA GGC
GAPDH-F	CCC CTT CAT TGA CCT CAA CTA CA
GAPDH-R	CGC TCC TGG AGG ATG GTG AT
TCRV-F	TCG GTC ACA GAT GGG ACC AGG
TCRV-R	CAG GGT TCT TCA CGT CCT CTG
ZIKV-F	CAG CAA TAG AGA CTT CGT GGA
ZIKV-R	CAG CAA TAG AGA CTT CGT GGA
hMYH9-F	CCT TCC GAC AAGAGT GGC TTT
hMYH9-R	GAG TAG TAA CGC TCC TTG AGG
mMYH9-F	CAT ACA ACA AAT ACC GCT TCC
mMYH9-R	TCA GTG TTC CGC TCC TTC TTG
GAP DNA-F	CCC CTT CAT TGA CCT CAA CTA CA
GAP DNA-R	CGC TCC TGG AGG ATG GTG AT
suMLV-F	CCT ACT ACG AAG GGG TGG
suMLV-R	CAC ATG GTA CCT GTA GGG GC
HSV1-TK-F	GAG TTT CAC GCC ACC AAG AT
HSV1-TK-R	CTA TGA TGA CAC AAA CCC CG
MAYV-F	AGG ACC CAG AGG AAC ACT AAT A
MAYV-R	CGA TAC TCT TTC GCC CAC TTA C

### Chemicals

Blebbistatin, ML-7, Y-27632, chlorpromazine, nystatin, 5-(N-ethyl-N-isopropyl)amiloride (EIPA), dynasore, PP1, piceatannol, wortmannin, and Jak inhibitor I were purchased from Sigma and reconstituted with DMSO or methanol following the manufacturer’s instructions. Adherent cells were pretreated with the inhibitors for 30–60 min, followed by viral infections for 1 h in the presence of the inhibitors. After the infection was completed, the cells were incubated without inhibitors for an additional 24 h.

### Generation of mouse primary cells

BMDMs were isolated from the hind limbs of 8- to 12-week-old C57BL/6 mice as previously described (3). Macrophages were cultured in DMEM complete supplemented with 100 µg/mL of macrophage colony-stimulating factor (M-CSF; Gibco) and 0.1% sodium pyruvate (Gibco). Cells were harvested 7 days after plating and seeded in 24-well plates for virus infections.

### *In vitro* and *ex vivo* infections

A549 cells were infected with TCRV, LCMV, ZIKV, and VSV at an MOI = 1 for 24 h, while HSV-1 infections (MOI = 1) were done in U2OS cells for 48 h. Viral nucleic acids were isolated at the indicated time points to assess infection levels by RT-qPCR (TCRV, LCMV, ZIKV, and VSV) or qPCR (HSV-1). MLV infections of 293T-mCAT cells were done at an MOI = 0.1 and viral DNA was measured by qPCR at 48 hpi. BMDMs were infected with TCRV and LCMV at an MOI = 1, and the cells were harvested at 48 hpi to assess infection levels by RT-qPCR.

### Western blot

Protein extracts (50 µg) were resolved on 10% SDS-polyacrylamide gels, transferred to polyvinylidene difluoride membranes (General Electric), and blocked with 3% bovine serum albumin (BSA; Sigma) or 5% non-fat milk. The following antibodies were used to detect endogenous, overexpressed, or fused proteins: rabbit anti-MYH9 (CST; #3403), rabbit anti-GAPDH (CST; #2118), mouse anti-FLAG (Invitrogen; # MA1-91878), rabbit anti-RLC (CST; #8505), rabbit anti-pRLC (Thr18/Ser19) (CST; #3674), mouse anti-Tubulin (Thermo; #A11126).

### Immunoprecipitation of endogenous proteins

A total of 500 µg of protein lysate per condition was incubated with a mix of rabbit anti-phospho-tyrosine antibodies (CST MultiMab; #8954), a rabbit monoclonal anti-MYH9 (Sigma; #M8064), or a rabbit IgG control polyclonal antibody (Proteintech; # 30000-0-AP) and 20 µL of Pierce Protein A/G agarose beads (Thermo; #20421) on rotation and at 4°C for 14 h. The immunocomplexes were then washed off five times with 1× cell lysis buffer and loaded onto SDS-polyacrylamide gels for western blot analysis.

### Nucleic acid isolation and RT-qPCR

DNA was isolated with the DNeasy Blood & Tissue Kit (Qiagen), and total RNA was isolated using the GeneJET RNA Purification Kit (Thermo). RNA was used as a template for cDNA synthesis using the RevertAid Reverse Transcriptase (Thermo) in a reaction mixture primed with 50 ng/µL of random hexamers (Macrogen). Viral and cellular RNAs were detected by RT-qPCR using a QuantStudio 3 Real-Time PCR System (Applied Biosystems) with specific primer pairs (Table 1), and the RNA expression was normalized to GAPDH. RT-qPCR reactions were done using Power SYBR Green Master Mix (Applied Biosystems), under these amplification conditions: 50°C for 2 min, 95°C for 10 min, and 40 cycles of 95°C for 15 s and 60°C for 1 min. The efficiency of amplification was determined for each primer set by a standard curve with 10-fold serial dilutions of DNA of known concentration. The slope values of the standard curves for the primer pair amplicons ranged from 3.5 to 3.2. For each primer pair, a non-template control was included, and each sample was run in triplicate.

### Confocal microscopy

A549 cells were infected with TCRV and LCMV at an MOI = 50 on ice for 1 h and then shifted to 37°C for different time intervals (0, 5, and 15 min). MYH9 sub-cellular localization and TCRV particles during virus entry were analyzed in a Zeiss LSM800 Confocal Microscope using a rabbit anti-MYH9 (Sigma; #M8064) and an anti-Junín nucleoprotein (clone IC06-BE10; BEI resources), and Alexa Fluor-conjugated (488 or 647) secondary antibodies (Invitrogen). Actin localization was determined using Texas Red-X Phalloidin (Invitrogen; #T7471).

### Virus entry assays

#### Binding assay

A549 cells were incubated on ice with TCRV or LCMV (MOI = 20) for 1 h, washed three times with 1× PBS to remove unbound particles, and total RNA was isolated to analyze genomic RNA levels of bound virions by RT-qPCR.

#### Internalization assay

A549 cells were infected on ice as described, washed with 1× PBS, shifted to 37°C for 45 min, treated with 1 mg/mL of Proteinase K (Gibco) for 45 min to strip off non-internalized particles from the cell surface, and treated with 2 mM of phenylmethylsulfonyl fluoride (PMSF) (Sigma) to inactivate Proteinase K. RNA was next isolated to analyze internalized viral RNA by RT-qPCR.

#### Fusion assay

A549 cells were co-transfected with a siMYH9 and a FLAG-tagged LCMV GP construct using Lipofectamine 3000 (Thermo) for 48 h. Next, transfected cells were pulsed with sodium citrate at pH = 5 or pH = 7 for 10 min, and the number and size of cell syncytia were determined by epifluorescence microscopy in four randomly sampled regions per condition, by analyzing the GP expression using an anti-FLAG antibody (Thermo).

### Generation of MYH9 mutant constructs

DNA constructs encoding full-length MYH9 wild-type (WT) (#11347) and the 3xA mutant (#101041) fused to GFP were purchased from Addgene. The 277F, 1805F and 2F constructs were generated by PCR-based site-specific mutagenesis using the MYH9 WT construct as template and the Q5 Site-Directed Mutagenesis Kit (New England Biolabs). The primers used to generate these mutants are detailed in Table 2. All introduced mutations were validated by Sanger sequencing (Macrogen).

**TABLE 2:** Primer pairs used for PCR-mediated mutagenesis^*a*^

Primer	Sequence (5’−3’)
MYH9-277F-For	$\text{CCA}\;\text{CAT}\;\text{CTT}\;\text{C}\frac{\text{TT T}}{\text{TA T}}\text{TA}\;\text{TCT}\;\text{CCT}\;\text{GTC}\;\text{TGG}\;\text{G}$
MYH9-277F-Rev	AAG GTC CGT TCT TCC TTG GCT
MYH9-1805F-For	$\text{CAA}\;\text{GTC}\;\text{CAA}\;\text{G}\frac{\text{TT T}}{\text{TA T}}\text{AA}\;\text{GGC}\;\text{CTC}\;\text{CAT}\;\text{CAC}\;\text{CG}$
MYH9-1805F-Rev	ACA GTG CCC TCC ATC TCC TGC

^*a*^ The primer positions are based on the MYH9 reference sequence CR456526.1. In bold is depicted the MYH9 sequence, with underlining indicating the coding sequences mutated from tyrosine to phenylalanine and bold indicating the unmutated codon.

### DNA transfection

DNA expression constructs were transfected into 80%–90% confluent cells using Lipofectamine 3000 (Thermo) for 24 h according to the manufacturer’s instructions. Cells were then lysed with 1× RIPA buffer supplemented with 1× Protease and Phosphatase Inhibitors (Thermo), the supernatants were clarified by centrifugation at 13,000 rpm and 4°C for 15 min, sonicated for 15 s, and stored at −70°C until use.

### Statistical analysis

Each experiment was done with three technical replicates per experiment. The data shown is the average of at least three independent experiments, or as indicated in the figure legends. Statistical analysis was performed using the GraphPad 8.1/PRISM software. Tests used to determine significance are indicated in figure legends.

## Data Availability

The data that support the findings of this study are available from the corresponding author upon request.
